# Latent Default Mode Network Connectivity Patterns: Associations With Sleep Health and Adolescent Psychopathology

**DOI:** 10.1002/brb3.70579

**Published:** 2025-05-19

**Authors:** Linhao Zhang, Charles Geier, Ellen House, Assaf Oshri

**Affiliations:** ^1^ Department of Human Development and Family Science University of Georgia Athens Georgia USA; ^2^ Department of Psychiatry and Health Behavior Augusta University Augusta Georgia USA; ^3^ University of Georgia Medical Partnership Athens Georgia USA

**Keywords:** externalizing problems, Fitbit, internalizing problems, resting‐state connectivity

## Abstract

**Background:**

The present study examined default mode network (DMN) neural connectivity patterns among adolescents. Next, we tested two critical markers of sleep health—duration and efficiency, in predicting neural connectivity patterns. Last, we investigated the latent DMN profiles’ predictive utility of internalizing and externalizing symptoms in youth.

**Methods:**

The study included 2811 youth (47.8% female; mean age = 11.94) enrolled in the Adolescent Brain Cognitive Development study. Sleep duration and efficiency were objectively measured via Fitbit wearable's (mean number of nights = 14.13). Latent profile analysis identified neural connectivity profiles within the DMN and between other networks (fronto‐parietal, salience, ventral attention, and dorsal attention). Parents reported the youth's psychopathology symptoms.

**Results:**

Four DMN profiles were empirically identified: (1) moderate; (2) low within and high between; (3) high within and low between; and (4) high within and high between. Youth with shorter sleep duration were more likely to be classified as low within and high between subgroup. Youth with lower sleep efficiency were more likely to be classified as the high within and low between subgroup. There were between‐group differences in externalizing problems one year later.

**Conclusion:**

Our findings highlight unique neural patterns in youth and their associations with sleep and psychopathology. The results will inform clinical practice and preventive programming that attempts to address the crisis in youth mental health through a focus on mitigating sleep problems in youth.

## Graphical abstract

Our findings reveal distinct neural patterns in youth linked to sleep and psychopathology, offering insights that can guide clinical practice and preventive interventions aimed at addressing the youth's mental health issues by focusing on improving sleep health.

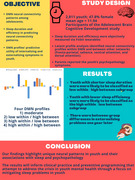

## 1 Introduction

During adolescence, neural functions undergo significant changes closely linked to psychopathology development (Casey et al. 2019; Casey et al. 2008). The DMN, a large‐scale functional brain network, significantly predicts psychopathology (Broyd et al. 2009). Importantly, higher‐order cognitive and emotional processes often depend not on a single network or isolated regions but on the connectivity of multiple networks (Benson et al. 2016; Bressler and Menon 2010). Despite this, we know little about individual differences in the communication between the DMN and other core networks. Further, sleep is a critical bio‐regulatory process (Galván 2020; Spencer 2013), facilitating neural restoration and growth as well as supporting cognitive, emotional, and behavioral functioning among youth. Growing research documents the neuroregulatory functions of the DMN and sleep (Galván 2020). Yet data on the associations between the DMN connectivity patterns, sleep health, and the developmental risk for psychopathology in adolescence remain alarmingly limited.

## 2 Neural Network Connectivity During Adolescence

Research on brain‐behavior associations has significantly advanced our understanding of the development of psychopathology (Casey et al. 2008). This body of work primarily focuses on particular brain regions and overlooks the importance of neural networks, which often govern complex behaviors (Pessoa 2014). There is also a gap in understanding how connectivity within and between neural networks collectively contributes to the development of psychiatric symptoms in youth. The connectivity among brain regions within a single network is called intra‐network connectivity or coherence (mean covariance across all regions). Inter‐network connectivity refers to the multivariate interactions between different functional brain networks (Tomasi and Volkow 2011). Examining both intra‐ and inter‐network connectivity provides a more comprehensive view of brain functioning by revealing how different neural networks interact. This approach allows us to more accurately predict specific behavioral responses based on the combined effects of these networks (Mooney et al. 2024).

The present study examines latent connectivity patterns within the DMN and its connectivity with other higher‐order executive networks. The DMN is a brain network characterized by a high level of resting metabolic activity, which typically decreases during cognitively demanding tasks (Sherman et al. 2014). Disruptions in connectivity between DMN regions are linked to deficits in decision‐making, goal‐directed behaviors, and cognitive inhibitions (Wade et al. 2018). DMN comprises multiple brain areas, including the anterior‐posterior midline regions of the medial prefrontal cortex (mPFC) (ventromedial PFC, anterior cingulate cortex), the medial parietal cortex (posterior cingulate cortex [PCC], precuneus, retrosplenial cortex) and lateral temporo‐parietal cortex (supramarginal gyrus, angular gyrus, and superior temporal sulcus) (Broyd et al. 2009). During adolescence, DMN exhibits significant development (Sherman et al. 2014). We focus on the DMN because it is consistently linked to neuroregulatory behaviors such as sleep (Tashjian et al. 2018), and risk for psychopathology (Whitfield‐Gabrieli and Ford 2012).

The DMN operates in tandem with core attention and emotion regulation brain networks rather than in isolation (Afzali et al. 2022; Ho et al. 2021; Yan et al. 2021). These networks include the frontal‐parietal network (FPN), dorsal attention network (DAN), ventral attention network (VAN), and salience network (SN). The FPN comprises the rostrolateral prefrontal cortex, middle frontal gyrus, anterior insula, dorsal anterior cingulate cortex, precuneus, caudate nucleus, dorsolateral prefrontal cortex, and the anterior inferior parietal lobule (Smallwood et al. 2012). FPN supports high‐level cognitive tasks critical for self‐regulation, including goal‐directed attention and working memory (Smallwood et al. 2012). DAN includes the intraparietal sulcus area and superior parietal lobule in posterior parietal cortex. The DAN activates when focusing attention on an object (Corbetta et al. 2008), and is thought to be responsible for goal‐directed, top‐down processing. VAN comprises the temporoparietal junction and the ventral frontal cortex. The VAN is generally activated when an unexpected event occurs and breaks one's attention from the current task (i.e., bottom‐up processing) (Corbetta et al. 2008). The SN, comprising the bilateral insula and anterior cingulate cortex, is a hub controlling the up regulation of attention networks and the down regulation of the DMN (Rosen et al. 2018). Together, these interconnected large‐scale neural circuits provide valuable insights into the organization and architecture of the brain crucial for data on clinical risk (Pessoa 2022).

## 3 Sleep Health and DMN Connectivity Patterns

Emerging neuroimaging research shows the association between behavioral sleep health and DMN functioning (Hehr et al. 2023; Tashjian et al. 2018). Tashjian et al. (2018) found that sleep quality (including efficiency), but not duration, was related to weaker within‐DMN connectivity in adolescents. A recent study using the adolescent brain and cognitive development (ABCD) dataset found that sleep duration, assessed via Fitbit wearable's, was associated with weaker within‐DMN resting‐state functional connectivity (rs‐FC) and higher rs‐FC between the DMN, DAN, and FPN (Hehr et al. 2023). These findings provided preliminary evidence that sleep duration and efficiency support brain functioning during a crucial neural and psychosocial development time (Telzer et al. 2013).

## 4 DMN Connectivity Patterns and Psychopathology

Disruptions in within‐and between‐DMN connectivity have been recognized to forecast psychiatric risk in youth (Afzali et al. 2022; Ho et al. 2021; Winters and Hyde 2022; Yan et al. 2021). Afzali et al. (2022) found that increases in depressive symptoms significantly predicted changes in functional connectivity between regions within the DMN. Adolescents with depressive symptoms also had lower connectivity in the anterior DMN and insula‐SN (Ho et al. 2021). In addition, individuals with impulsive behavior, such as online gambling exhibited increased connectivity between the DMN and VAN (Yan et al. 2021). Collectively, these studies suggest that DMN connectivity indicators, such as within‐DMN and DMN‐VAN connectivity, independently emerge as robust predictors of psychiatric symptoms. However, less data exists on how these connectivity patterns interact to shape psychiatric outcomes. Investigating these relationships presents a methodological challenge, given the significant variations in connectivity within the DMN and other neural networks during adolescence (Gozdas et al. 2019). The complexity and variability in the brain could manifest in different subpopulations with unique connectivity patterns, necessitating a robust multivariate statistical approach.

## 5 A Latent Pattern Methodological Approach

Latent profile analysis (LPA) is a pattern‐based analytical tool that identifies individuals’ unobserved latent patterns based on multivariate similarities (Muthén and Muthén 2000). Utilizing a network pattern approach to understanding neural function acknowledges the brain's interconnected and dynamic nature and provides a more comprehensive view than examining singular networks alone. Specifically, LPA moves beyond studying individual network functions and identifies latent within and between connectivity patterns. Using LPA is advantageous because it models continuous measures and captures the variability of each examined network connectivity indicator. Previous neuroimaging LPA studies have presented uniform evidence regarding the associations between neural patterns and psychopathology. Huffman et al. (2022) identified three profiles that were primarily differentiated by neural response to emotional stimuli within the amygdala, anterior cingulate cortex, and insula across early adolescence. No associations were found between the development of neural function and psychopathological symptoms. On the other hand, Fekson et al. (2023) showed four inhibitory control groups and significantly different reading abilities, with neurobiological differences. However, to our knowledge, no study has comprehensively documented latent DMN connectivity patterns using five major brain networks and their association with sleep and psychopathology in a large and longitudinal sample of youth.

## 6 Present Study

We first aim to identify patterns of DMN network connectivity using multiple indicators (within‐DMN, DMN‐FPN, DMN‐VAN, DMN‐DAN, and DMN‐SN connectivity; (Aim 1). We then examine whether sleep duration and efficiency (measured objectively via Fitbit wearable's), as well as demographic covariates (e.g., age, sex, and minority status), are associated with DMN profile memberships cross‐sectionally (Aim 2). Third, we test the predictive utility of brain network connectivity profiles for the development of internalizing and externalizing symptoms cross‐sectionally and one year later (Aim 3).

## 7 Methods

### 7.1 Sample

All hypotheses were tested using data from the ABCD Study (5.0 data release), an ongoing, multi‐site longitudinal study of adolescent brain development and mental health (Casey et al. 2018). Institutional review boards approved all study procedures, with consent obtained from caregivers and youth (Casey et al. 2018). The present study began its analysis in Year 2, as this was when Fitbit data became available. We utilized data from Year 2 (47.8% female; mean age = 11.94), including Fitbit, neuroimaging, and psychopathology measures, and Year 3, which provided additional data on psychopathology symptoms. The final analysis comprised 2811 youth with both MRI and Fitbit sleep data (out of 6793 participants with MRI data, and 3326 participants with more than seven nights of Fitbit sleep data). The sample's racial‐ethnic composition was 52.0% white, 15.0% black, 20.3% hispanic, 2.1% asian, and 10.5% other.

### 7.2 Imaging Data

All neuroimaging data were collected on Siemens, General Electric, or Philips 3T scanners with a 32‐channel head coil. The participants viewed a crosshair for approximately 20 min during the rs‐FC MRI data acquisition while awake with their eyes open. Scanning parameters and protocols for each scanner type and all sequences were provided elsewhere (Hagler et al. 2019). Briefly, rs‐FC MRI data were preprocessed using FreeSurfer version 5.3.0 (Casey et al. 2018) by the ABCD Data Analysis and Informatics Core (Hagler et al. 2019). Rs‐FC analysis was computed using a seed‐based, correlational approach; see Hagler et al. (2019) for detailed data processing procedures. We followed the ABCD quality control protocol and only included participants with > 12.5 min of data with frame wise displacement (FD) < 2 mm at any direction (x, y, z, pitch, roll, and yaw) (Hagler et al. 2019).

### 7.3 Measures

#### 7.3.1 DMN rsFC

Network connectivity was calculated using Pearson correlation based on the Gordon parcellation scheme for predefined resting‐state networks in Year 2 (Gordon et al. 2016). We included within‐DMN and DMN‐FPN, DMN‐SN, DMN‐DAN, and DMN‐VAN connectivity. Within network correlation was calculated as the average Fischer *r*‐to‐*Z* correlations for each pairwise combination of regions of interest that belong to each network (e.g., DMN). The average correlation between one network and another was calculated similarly by averaging the correlations for each unique, pairwise combination of ROIs in the first network with the ROIs in the second. Supplementary Figure 1 includes brain regions in each brain network. A higher score meant a higher average correlation over all pairs of regions within the DMN or higher connectivity between the DMN and other networks. Additional methodological information can be found in Casey et al. 2018 and Hagler et al. 2019.

#### 7.3.2 Sleep

Adolescents wore Fitbit charge HR watches for up to 21 nights to measure their sleep in Year 2. Fitbit has been validated against research‐grade, gold‐standard devices in developmental samples of children and adolescents (Godino et al. 2020). Participants with at least seven nights of sleep data were included (mean day count = 14.13; standard deviation of day count = 3.98). The ABCD data team conducted preliminary cleaning and scoring. Then, weighted weekday‐weekend sleep duration and efficiency (percentage of minutes scored asleep) were calculated ((e.g., (weekday sleep duration X weekday day counts) + (weekend sleep duration X weekend day counts))/ (total day counts)) and winsorized (‐ + 2*SD*) (Ghosh and Vogt 2012). A weighted score can potentially avoid results driven by either predominantly weekday or weekend sleep data and capture a more overall sleep health (Zhang et al. 2023).

#### 7.3.3 Psychopathology

Primary caregivers reported on youth internalizing and externalizing symptoms in Year 2 and Year 3 using the child behavior checklist (Bilenberg 1999) to test cross‐sectional and longitudinal associations. 119 items were assessed on a scale ranging from “0” (not true) to “2” (very true). The alphas of internalizing (α_two‐year_ = 0.88; α_three‐year_ = 0.89) and externalizing problems (α_two‐year_ = 0.90; α_three‐year_ = 0.90) were acceptable.

#### 7.3.4 Demographic Covariates

In addition to sleep duration and efficiency, we tested whether demographic covariates—youth sex (1 = female, 2 = male), age, and minority status (white = 0, racial minority [e.g., black, hispanic, asian, and other] = 1) were significant predictors of profile membership.

### 7.4 Analytic Plan

Analysis were performed in Mplus 8.2. LPA was used to determine DMN connectivity profiles in Year 2 (Aim 1). The optimal class solution was determined through conceptual considerations and model fit criteria (Lubke and Muthén 2005), including akaike's information criterion (AIC), bayesian information criterion (BIC), sample‐size adjusted BIC (SABIC), Lo‐Mendell‐Rubin adjusted test (LMRT), and entropy. Decreases in AIC, BIC, and SABIC indicate a better model fit than the previous model. A significant *p*‐value of LMRT indicates that the target model has a significantly better model fit than the model with one profile less. Entropy values nearing 1 indicate clear profile delineation, with a cutoff between 0.60 and 0.80 as appropriate (Spurk et al. 2020). The profile classification was tested in (1) full MRI data participants (*n* = 6793); and (2) a final analytical sample with both MRI data and Fitbit sleep data (*N* = 2811).

We then tested how sleep duration, efficiency, and demographic covariates (sex, age, and minority status) associated with DMN rs‐FC profile memberships (Aim 2). Specifically, the four profiles were regressed onto sleep indicators and demographic covariates. These multinomial logistic regressions yield odds ratios and indicate the odds of belonging to a latent profile given a predictor variable. Third, we examined the concurrent and prospective links between profile membership and each outcome (Aim 3). To assess the predictive utility of the profiles, we conducted linear regression using the Bolck, Croon, and Hagenaars (BCH; 2004) correction method to estimate between‐group mean differences in youth internalizing and externalizing problems, both concurrently and one year later (four outcomes) within a single model. Aims 2 and 3 were tested using a more rigorous sample of participants with both MRI and Fitbit sleep data (*N* = 2811) to minimize the overestimation of missing Fitbit data. Other missing data on psychopathology symptoms at a later time point (Year 3; 10.08%) were estimated using a full information maximum likelihood algorithm and hypotheses were tested using maximum likelihood estimation with robust standard errors. Multilevel modeling (Mplus clustering command) and propensity weights were used to account for the clustering effects (within families and sites) and mitigate potential selection bias in the ABCD recruitment process. Given that the ABCD dataset included oversampled siblings and twins across 21 sites in the U.S., it was essential to consider the nested structure of individuals within families and sites (Saragosa‐Harris et al., 2022).

## 8 Results

### 8.1 Preliminary Analyses

Descriptive statistics and bivariate correlations among study variables were presented in Table 1. The average sleep duration was 7.5 h, with a sleep efficiency of 89%. Sleep duration was associated with higher sleep efficiency, higher within‐DMN connectivity, and lower DMN‐FPN and DMN‐DAN connectivity. Both sleep duration and efficiency were negatively associated with externalizing problems in Year 2 and Year 3 and were not associated with internalizing problems in Year 2 and Year 3. Youth with minority status, older age, and males were associated with shorter sleep duration. White and male youth were more likely to have lower sleep efficiency. Youth with minority status were more likely to be associated with externalizing problems in Year 2 and Year 3. Males were more likely to be associated with externalizing problems in Year 2 and Year 3 and internalizing problems in Year 2.

**TABLE 1:** Bivariate correlations, mean, and standard deviation among study variables (*N* = 2811).

	1	2	3	4	5	6	7	8	9	10	11	12	13	14
1.Within‐DMN Year 2	__													
2.DMN‐FPN Year 2	0.11^**^	__												
3. DMN‐SN Year 2	0.19^**^	0.27^**^	__											
4. DMN‐VAN Year 2	0.37^**^	0.12^**^	0.22^**^	__										
5. DMN‐ DAN Year 2	−0.69^**^	0.20^**^	−0.03	−0.29^**^	__									
6. Sleep duration Year 2	0.11^**^	−0.05^**^	0.00	0.10^**^	−0.12^**^	__								
7. Sleep efficiency Year 2	−0.01	0.01	−0.01	−0.03	0.03	0.06^**^	__							
8. Externalizing problems Year 2	−0.04^*^	0.03	0.01	−0.01	0.05^**^	−0.05^**^	−0.04^*^	__						
9. Internalizing problems Year 2	−0.01	−0.01	0.01	−0.02	0.01	−0.01	−0.02	0.58^**^	__					
10. Externalizing problems Year 3	−0.07^**^	0.01	0.02	−0.03	0.09^**^	−0.06^**^	−0.04^*^	0.72^**^	0.46^**^	__				
11. Internalizing problems Year 3	−0.005	0.01	0.02	0.00	0.01	−0.001	−0.003	0.44^**^	0.69^**^	0.59^**^	__			
12. Youth minority status	−0.13^**^	0.06^**^	−0.05^**^	−0.11^**^	0.15^**^	−0.20^**^	0.06^**^	0.04^*^	0.01	0.04*	−0.01	__		
13. Youth age Year 2	0.04^**^	−0.03	−0.02	−0.04	−0.06^**^	−0.13^**^	−0.002	−0.04*	−0.05^**^	−0.06^**^	−0.06^**^	−0.03	__	
14. Youth sex	−0.19^**^	0.01	−0.08^*^	−0.11^**^	0.14^**^	−0.09^**^	−0.08^**^	0.08^**^	0.05^**^	0.07^**^	−0.02	−0.01	0.002	__
Mean	0.25	0.05	0.07	0.08	−0.14	7.50	0.89	43.96	47.72	44.07	47.85	0.38	11.92	0.51
Standard deviation	0.06	0.04	0.06	0.05	0.06	0.56	0.03	9.47	10.20	9.38	10.45	0.49	0.64	0.50

*Note*: DMN = default mode network; FPN = frontal‐parietal network; SN = salience network; VAN = ventral attention network; DAN = dorsal attention network. Youth minority status coded as 0 = white, 1 = race‐ethnic minority; youth sex coded as 0 = female, 1 = male. Externalizing and internalizing problems were measured using CBCL T‐scores and the clinical cut off is 64. There were 2.9% participants in Year 2 and 3.1% in Year 3 met clinical cut off for externalizing problems. There were 7.2 % participants in Year 2 and 8.1% in Year 3 met clinical cut off for internalizing problems. *p < 0.05, **p < 0.01, ***p < 0.001.

### 8.2 Classification and Memberships

The AIC, BIC, and SABIC decreased, indicating that the four‐class, five‐class, and six‐class solutions fit the data better than the three‐, two‐, and one‐class solutions. However, we did not select the six‐class model because the Lo‐M‐R ALRT yielded a *p*‐value of 0.05. While the four‐class and five‐class solutions had similar entropy, the smallest class in the five‐class solution only comprised 6.26% of the sample (*n* = 176), compared to 11.51% (*n* = 324) in the four‐class solution, nearly twice the size. The decrease in BIC from the four to five classes was not substantial compared to the drop from three to four classes (Table 2). Scree plots were used to identify the optimal number of classes (Nylund‐Gibson and Choi 2018). The “elbow” in the plot, indicating the best‐fitting solution, appeared at the four‐profile solution (see Supplementary Figure 2). Therefore, a four‐class solution was selected.

**TABLE 2:** Model fit indices of different profile solution.

Profile solution number	Lo‐M‐R ALRT	*p*	AIC	BIC	BIC‐Adj.	△ BIC‐Adj	Entropy	Smallest profile (%)
Two‐profiles	1458.74	<0.001	38426.90	38521.96	38471.13	—	0.67	1105 (39.30)
Three‐profiles	479.92	0.003	37948.91	38079.62	38009.72	561.41	0.71	434 (15.45)
Four‐profiles	344.25	0.002	37609.44	37775.80	37686.83	322.89	0.68	324 (11.51)
Five‐profiles	250.66	0.04	37365.52	37567.53	37459.50	227.33	0.70	176 (6.26)
Six‐profiles	148.61	0.05	37225.79	37463.44	37336.35	123.15	0.71	176 (6.26)

*Note*: BLRT = bootstrapped likelihood ratio test; Lo‐M‐R ALRT = Lo–Mendell–Rubin Adjusted Likelihood Ratio Test; AIC = Akaike information criterion; BIC = Bayesian information criterion. BIC‐adj. = sample size–adjusted BIC. △BIC‐ Adj = change in adjusted BIC from the *k*‐1 class to the *k* class.

**FIGURE 1:**
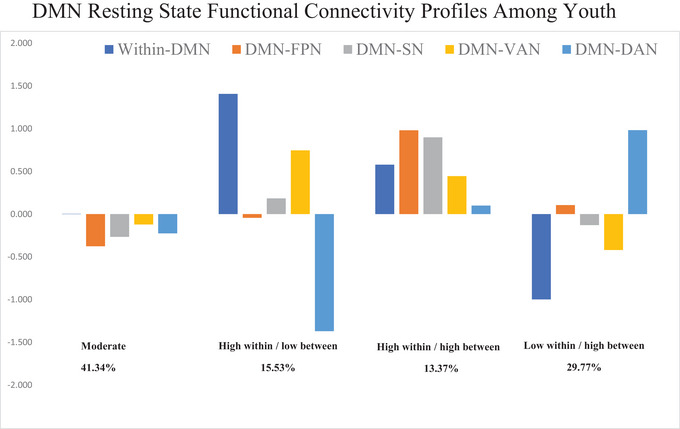
*Notes*. DMN resting state functional connectivity profiles among youth. Connectivity parameters values are centered around the mean (i.e., zero); *N* = 2811; DMN = default mode network; FPN = frontal‐parietal network; SN = salience network; VAN = ventral attention network; DAN = dorsal attention network.

Profile analyses were performed on full MRI data participants (*n* = 6793), and the final analytical sample (*n* = 2811), and profile characteristics were consistent. Thus, we focused on a more rigorous subset of individuals with both Fitbit and MRI data (*n* = 2811) here to avoid overestimating missing values for subsequent analyses. Four subgroups were identified based on rs‐FC within the DMN and its connectivity with other large networks. There were five indicators including within‐DMN, DMN‐FPN, DMN‐SN, DMN‐VAN, and DMN‐DAN connectivity. The LPA brain connectivity indicators were standardized (z‐scored) and the four‐profile solutions were presented in Figure 1. Profile 1 (*n* = 1212; 43.12%) was characterized by average within‐ and between‐DMN connectivity with other networks. We labeled profile 1 as the *moderate* subgroup. The second largest profile (*n* = 835; 29.30%) was characterized as the lowest within‐DMN connectivity, below‐average DMN‐VAN, average DMN‐FPN, average DMN‐SN, and highest DMN‐DAN connectivity. This group was named as the *low within and high between* subgroup. The third profile (*n* = 441; 15.68%) was characterized by the highest within‐DMN connectivity and average DMN‐SN, average DMN‐FPN, above‐average DMN‐VAN, and lowest DMN‐DAN connectivity. We named this group as *high within and low between* subgroup. The smallest profile (*n* = 324; 11.51%) was characterized as high within‐DMN, DMN‐FPN, DMN‐SN, DMN‐VAN, and DMN‐DAN connectivity. This group was named the *high within and high between* subgroup (Figure 1). The reference group was *low within/ high between groups* because this profile was the only group with lower‐than‐average DMN.

### 8.3 Associations With Profiles

Sleep duration, efficiency, and other demographic covariates were examined in the same model. As such, each predictor was independently associated with DMN profile membership, controlling for other predictors in the model. Youth with shorter sleep duration were more likely to be classified as *low within and high between* subgroup than the other three subgroups. Youth with lower sleep efficiency were more likely to be classified as *high within and low between* subgroup than *low within / high between* subgroup. Male youth were more likely to be classified in the *low within and high between* subgroup compared to the other three groups. Additionally, youth who identified as a minority were more likely to be classified as *low within and high between* subgroup than the *moderate* and *high within and low between* groups. However, no significant age differences were observed across profiles (Table 3).

**TABLE 3:** Multinomial logistic regression results comparing DMN profiles by covariates. Reference profile: Low within and high between.

Profile	Covariate	Logit	SE	OR
Moderate	Sleep duration	0.35	0.11	1.41^**^
	Sleep efficiency	0.10	3.02	1.11
	Youth minority status	−0.59	0.15	.55^***^
	Youth age	0.25	0.14	1.28
	Youth sex	−0.48	0.15	.62^***^
High within and low between	Sleep duration	0.44	0.18	1.56^*^
	Sleep efficiency	−6.04	3.14	.002^***^
	Youth minority status	−0.78	0.18	.46^***^
	Youth age	0.26	0.17	1.30
	Youth sex	−1.13	0.17	.32^***^
High within and high between	Sleep duration	0.50	0.18	1.64^*^
	Sleep efficiency	2.31	4.28	10.10
	Youth minority status	−0.30	0.21	.74
	Youth age	−0.07	0.20	.93
	Youth sex	−1.11	0.21	.33^***^

*Note*: Logit = logistic regression; OR = odds ratio; youth minority status coded as 0 = white, 1 = race‐ethnic minority; youth sex coded as 0 = female, 1 = male. *p < 0.05, **p < 0.01, ***p < 0.001.

### 8.4 Latent Profiles and Psychopathology

Youth in the *low within and high between* subgroup were more likely to report externalizing problems in Year 3 than those in the *high within and low between* subgroup (Table 4). After multiple comparison tests, the corrected *p*‐value was 0.024. We did not see other significant group differences.

**TABLE 4:** Means (standard deviation) of outcomes across profiles.

Outcomes	Moderate	High within and low between	High within and high between	Low within and high between
Externalizing problems at Year 2	44.04 (0.42)	43.72 (0.64)	44.99 (0.86)	45.11 (0.48)
Internalizing problems at Year 2	48.13 (0.45)	47.86 (0.71)	47.80 (0.94)	47.85 (0.51)
Externalizing problems at Year 3	44.13 (0.44)	42.97 (0.58)	44.83 (0.88)	45.39 (0.46)
Internalizing problems at Year 3	48.43 (0.48)	47.75 (0.69)	48.44 (0.96)	47.82 (0.50)

*Note*: Significant results are bolded. There is a group difference between the *high within and low between* subgroup and the *low within and high between* subgroup (*p* < 0.001).

## 9 Discussion

Sleep health is critical for adolescent brain development and mental health (Galván 2020). Sleep behaviors change considerably during adolescence and significantly influence psychiatric risk (El‐Sheikh et al. 2019). The present study documented the DMN profiles and their associations with sleep health and risk for the development of psychopathology. In a large prospective sample of youth, we identified four neural profiles based on DMN intra‐ and inter‐network connectivity: (1) *moderate*; (2) *low within and high between*; (3) *high within and low between*; and (4) *high within and high between*. Adolescents in the *low within and high between* subgroup had shorter sleep duration. Lower sleep efficiency was found to be associated with youth who were classified as *high within and low between* profile. We also found between‐profile differences in the development of externalizing problems in Year 3.

This study used LPA to identify DMN connectivity patterns among early adolescents. Our results showed that three of four profiles were characterized by above‐average within‐DMN connectivity. Theseheightened DMN rs‐FC patterns aligned with previous data showing normative increases in connectivity within networks during early adolescence (Gard 2021; Sherman et al. 2014). Notably, our neural connectivity patterns revealed significant variability within the DMN and in the connectivity between the DMN and attention networks among adolescents. This variability allowed us to classify these profiles as “*high within and low between*,” indicating high connectivity within the DMN and low connectivity between the DMN and the DAN, or as “*low within and high between*,” reflecting low connectivity within the DMN and high connectivity between the DMN and the DAN. Overall, a pattern‐based approach emphasized the advantage of focusing on latent brain activation typologies, offering a deeper understanding of individual variability in neural functioning and its implications for cognitive and behavioral outcomes. Our findings informed interventions to target strategies to address the heterogeneity and move beyond one‐size‐fits‐all neuroscience models. Future research could investigate, replicate, and build upon our framework to establish rs‐FC patterns with other core networks in youth.

Our findings indicated that male and minoritized adolescents were more likely to be classified in the *low within and high between* network connectivity subgroup. This subgroup was also more likely to show shorter sleep duration and increased externalizing problems. These results aligned with prior research demonstrating demographic differences in neural activity and connectivity. For instance, Lee et al. (2024) found that changes in externalizing problems were predicted by hyper connectivity between core DMN nodes and the front parietal network in boys, whereas girls exhibited hypo connectivity between the DMN and affective networks. Additionally, research by Mathur et al. (2012) suggested that racial identification influenced how individuals recruit neural and cognitive processes when interacting with people from their own and other racial groups. These findings highlight the importance of developing targeted interventions addressing DMN connectivity based on gender, race, and cultural factors to support adolescent brain development and mental health. Future research could also explore how sex and minority status may moderate the association between DMN profiles and behavioral outcomes.

We found that youth with the shortest sleep duration were more likely to show DMN connectivity patterns, as observed in the *low within and high between* subgroup. In healthy individuals, DMN typically exhibited strong rs‐FC within‐network and anti‐correlation with task‐positive networks (e.g., attention, FPN, and SN) (Hehr et al. 2023). Insufficient sleep could potentially disturb the brain's balance of cognitive processes and the dynamic interplay between internally directed processes (within‐DMN) and externally directed attention‐demanding tasks (Brooks et al. 2022). This disruption may manifest as increased connectivity with attention networks, possibly stemming from diminished efficiency in communication among brain areas. Such heightened between‐network connectivity might reflect an effort to compensate for the disrupted patterns within the DMN (Brooks et al. 2022). Our findings showed that sleep duration was associated with DMN connectivity patterns, even in a community sample without severe sleep deprivation (Hehr et al. 2023).

Sleep is a multi‐faceted construct. While previous research, such as Hehr et al. (2023), explored the impact of sleep duration and disturbance on neural network connectivity, our study incorporated sleep efficiency, highlighting its unique contribution to neural network connectivity patterns. Specifically, youth with lower sleep efficiency were more likely to be classified as *high within and low between* than *low within and high between* subgroup. Although our results did not support previous research showing that poor sleep quality (including sleep efficiency) was linked to lower within‐DMN connectivity (Tashjian et al. 2018), our findings converge with data on the connection between poor sleep quality and low rs‐FC DMN and FPN (between network connectivity). Notably, the *low within and high between* subgroup had higher sleep efficiency yet the lowest sleep duration. The higher sleep efficiency may partially be compensated for the sleep loss in this subgroup. The compensatory theory of sleep suggests that when sleep quantity is reduced, the body compensates by enhancing sleep efficiency or other restorative processes. This adjustment helps to alleviate some of the negative effects of insufficient sleep, though it may not completely offset the long‐term consequences of chronic sleep deprivation (Sadeh and Gruber 2002). By collecting sleep duration and efficiency for up to 21 days via Fitbit wearable's, our study extended from studies that relied on single‐point self‐report assessments like questionnaires. Considering both sleep duration and efficiency were essential for promoting overall sleep health during adolescence.

The identified DMN profiles evinced differential risk for psychopathology. One innovation of the present study was to examine internalizing and externalizing problems concurrently and longitudinally over one year. Specifically, the *low within and high between* subgroup had the highest externalizing problems in Year 3. Previous research showed that lower connectivity within‐DMN and higher DMN‐DAN connectivity were widely observed in individuals with ADHD, attention problems, and callous‐unemotional traits (Owens et al. 2020; Umbach and Tottenham 2021). However, we only observed group differences for externalizing problems in Year 3, not Year 2. This may be due to the changing vulnerability over time to the development of psychopathology in adolescence (mean age of Year 2 = 11.94 to mean age of Year 3 = 12.89). The non‐significant results may also be due to the time required for neural risk factors to manifest in psychopathology. Future research should continue to investigate the longitudinal impact of neural connectivity patterns on the development of psychopathology when more data is available in the ABCD study.

No significant group differences were observed in the development of internalizing problems at either the Year 2 or Year 3 data collection. Our results diverged from prior research that underscored the involvement of DMN patterns in risk for psychopathology (Bosch et al. 2013; Chahal et al. 2021). Additionally, our results contrasted with studies highlighting the variations in connectivity between the DMN, FPN, and DAN among youth experiencing internalizing problems (Albertina et al. 2022). The lack of significant findings may be due to the sample, which consisted of early adolescents from community settings, with only 8.1% meeting clinical cutoff criteria. Future research should extend this investigation to clinical settings to test our hypotheses further.

## 10 Limitations and Future Directions

There were limitations in the present study. First, Fitbit wearable's may have limitations compared to polysomnography, the “gold standard” sleep measurement. To ensure the reliability of our analysis, we selected participants who had seven or more days of Fitbit data. Future research could also assess other sleep indicators (e.g., daytime naps) and validate our findings by adding sleep diaries or polysomnography. Second, objective sleep measures utilized in the present study provided valuable insights into the associations between sleep duration, efficiency, brain network connectivity, and behavioral problems. However, it is important to consider rapid eye movement (REM) and non‐REM sleep as sleep architecture undergoes significant developmental maturation during adolescence. Non‐REM sleep has been associated with synaptic pruning, brain volume, and internalizing problems (Akbar et al. 2022; Blake et al. 2018). Future research should examine how different sleep stages influence adolescent development, as this could provide targeted implications for fostering healthy sleep patterns and improving behavioral outcomes. Third, the directionality of the sleep‐to‐brain associations cannot be established, as full Fitbit data was only available in Year 2. Additional time points are needed to accurately assess the direction of these associations. Fourth, this study utilized preprocessed imaging data and was limited to mean network connectivity values. Future research should expand upon our hypotheses by examining sub‐regions within DMN to identify distinct sub region connectivity within the DMN. Fifth, this study examined five major brain networks, offering valuable insights into how higher‐order networks contribute to adolescents’ externalizing problems. Future research could build on these findings by incorporating additional core brain regions or networks and providing a more comprehensive understanding of individual differences and whole brain functioning in relation to behavioral outcomes. Sixth, although study sites were controlled for, regional variability across different parts of the U.S. may have systematically influenced differences in data collection and participant responses, which should be taken into consideration.

Despite these limitations, this was the first study to document how adolescent sleep patterns were related to connectivity across five major brain networks and their implications for psychiatric risk. In particular, the study shed light on the associations between behavioral sleep data, functioning, and risk for psychopathology problems. Our findings suggested that interventions targeting improved sleep health and brain network connectivity could help mitigate risks for psychopathology problems in adolescence and adulthood.

## Author Contributions

**Linhao Zhang**: writing–original draft, conceptualization, methodology, visualization, data curation, formal analysis, writing–review and editing. **Charles Geier**: methodology, writing–original draft, writing–review and editing, supervision, conceptualization. **Ellen House**: writing–review and editing, writing–original draft, conceptualization. **Assaf Oshri**: conceptualization, supervision, writing–original draft, writing–review and editing, formal analysis, methodology.

### Peer Review

The peer review history for this article is available at https://publons.com/publon/10.1002/brb3.70579

## Supporting information




**Supplementary Figure 1**: Brain regions from the five networks included in the analysis.
**Supplementary Figure 2**: Scree plot for decrease in BIC for different numbers of profiles.

## Data Availability

The data that support the findings of this study are available from the corresponding author upon reasonable request.
