# Supplementary material for: Latent Default Mode Network Connectivity Patterns: Associations With Sleep Health and Adolescent Psychopathology
Source: Brain Behav. 2025 May 19;15(5):e70579. doi: 10.1002/brb3.70579 (PMC12086304; doi:10.1002/brb3.70579)
Supplement: Supplementary file 1 — Supplementary Figure 1: Brain regions from the five networks included in the analysis. Supplementary Figure 2: Scree plot for decrease in BIC for different numbers of profiles. [file BRB3-15-e70579-s001.docx]

**Supplementary Figure 1.**

Brain regions from the five networks included in the analysis.
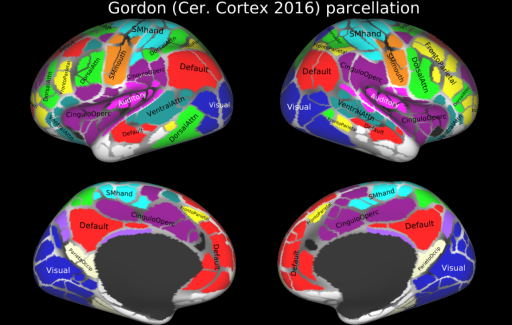


Note: The Salience Network was labeled as “Cingulo-Opercular” in the graph.

**Supplementary Figure 2.**

Scree plot for decrease in BIC for different numbers of profiles. **
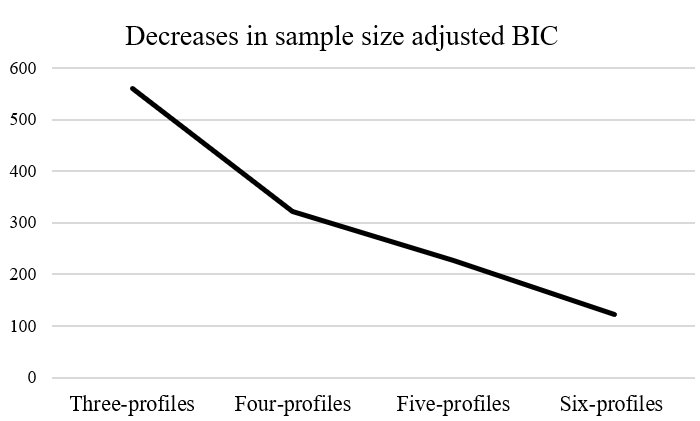
**
